# Engagement in binge eating and fasting associated with poorer sleep quality in an online sample of adults

**DOI:** 10.1186/s40337-024-01028-6

**Published:** 2024-06-10

**Authors:** Leah A. Irish, Kara A. Christensen Pacella, Maegan B. Nation, Rachel D. Barnes

**Affiliations:** 1 Department of Psychology, North Dakota State University, Fargo, ND USA; 2 Center for Biobehavioral Research, Sanford Research-North, Fargo, ND USA; 3 Department of Psychology, University of Nevada Las Vegas, Las Vegas, NV USA; 4 Department of Psychology, University of Pittsburgh, Pittsburgh, PA USA; 5 Health Psychology General Internal Medicine, University of Minnesota Medical School, MMC 741, 420 Delaware Street SE, Minneapolis, MN 55455 USA

**Keywords:** Sleep quality, Binge eating, Fasting, Compensatory behaviors, Purging

## Abstract

**Objectives:**

Both disordered eating and disturbed sleep represent significant threats to mental health. Accumulating evidence suggests that disordered eating behaviors and sleep problems co-occur. A majority of current research, however, has focused on these behaviors as components of eating disorder diagnoses, rather than investigating the independent associations of transdiagnostic disordered eating behaviors and sleep. The present study sought to examine fasting, binge eating, self-induced vomiting, laxative or diuretic misuse, and driven exercise as predictors of sleep quality complaints.

**Method:**

An online sample of 648 U.S. adults completed the Eating Disorder Examination - Questionnaire, the Pittsburgh Sleep Quality Index, and the Patient Health Care Questionnaire-2 as part of a larger parent study.

**Results:**

Results of a hierarchical linear regression revealed that, collectively, disordered eating behaviors predicted worse sleep quality [R^2^ = 0.30, *F*(7, 640) = 31.21, *p* < .001], and that both binge eating and fasting, but not other compensatory behaviors, accounted for unique variance in sleep quality after controlling for BMI and depression.

**Conclusions:**

Overall, findings suggest that transdiagnostic disordered eating behaviors are associated with sleep quality complaints. Improved understanding of the specific relationships between individual eating behaviors and sleep characteristics can help refine the identification of individuals at high risk for sleep disturbance and address the potential reciprocal influence of sleep quality on disordered eating behaviors.

**Supplementary Information:**

The online version contains supplementary material available at 10.1186/s40337-024-01028-6.

## Abstract (plain language summary)

Both disordered eating and problematic sleep negatively impact mental health. Many times they occur together and it is likely that worse sleep may intensify disordered eating symptoms and vice versa. Previous research tends to be limited to assessing sleep based on the presence or absence of an eating disorder diagnosis, rather than exploring associations between sleep and different types of eating disorder behaviors, such as fasting, binge eating, self-induced vomiting, laxative or diuretic misuse, and driven exercise. Thus, the current study examined symptoms of eating disorders that may occur across different eating disorder diagnoses and how they relate to sleep quality complaints. Six hundred forty-eight adults completed online questionnaires related to sleep, eating, and depression. Results of the current study found that higher weight, depression, fasting, and binge eating were most strongly related to worse sleep quality. Eating disorder symptoms such as binge eating and fasting may occur across eating disorder diagnoses, suggesting the importance of examining eating disorder behaviors, rather than diagnoses only. Continued understanding of particular risk factors for sleep problems within disordered eating will help identify high risk individuals who may need additional support for both problematic eating and sleep.

**Supplementary Information:**

The online version contains supplementary material available at 10.1186/s40337-024-01028-6.

## Introduction

Eating disorders have a lifetime prevalence of 9% among American adults [1] and confer increased mortality risk, medical and psychiatric comorbidities, and financial burden [1, 2]. Subclinical eating disorders (i.e., engaging in clinically significant levels of disordered eating in the absence of full diagnostic criteria) are much more common in the general population than diagnostic eating disorders, but associated with similar adverse health outcomes and functional impairment [3–8]. Sleep problems are also common among American adults [9, 10] and are associated with increased risk for negative physical and mental health outcomes [11, 12]. Disordered eating and sleep problems often co-occur. For example, Kim and colleagues reported that 50.3% of women with eating disorders reported sleep disturbances, and that these sleep disturbances were associated with greater frequency of disordered eating behaviors [13].

Recent literature reviews and a meta-analysis have supported the empirical link between sleep and disordered eating behaviors [14–16]. The nature of this relationship is likely bidirectional, such that sleep disturbance increases the risk of disordered eating behavior and, in turn, disordered eating behavior increases the risk of disturbed sleep [17]. To date, research investigating the link between disordered eating and sleep has focused largely on comparing sleep characteristics between individuals diagnosed with eating disorders (e.g., anorexia nervosa, bulimia nervosa) and healthy controls or correlating global eating disorder psychopathology with sleep quality [14, 15]. However, a number of key disordered eating behaviors occur across multiple diagnostic categories [18], such as objective binge eating (i.e., consuming an unusually large amount of food in a discrete period of time accompanied by a subjective loss of control), fasting (i.e., not eating for an extended period of time for the purpose of influencing body shape or weight), and other compensatory behaviors (i.e., self-induced vomiting, use of diuretics or laxatives, or driven exercise).

In a recent review, Christensen & Short [17] proposed a number of biobehavioral mechanisms that may explain the bidirectional relationship between sleep disturbance and disordered eating behaviors. For example, this model suggests that insufficient sleep likely alters appetitive behaviors through increases in food craving and alterations in levels of leptin and ghrelin which may result in increased feelings of hunger and decreased feeling of satiety. Insufficient sleep also results in impaired emotion regulation and cognitive functions thus increasing the likelihood of engaging in disordered eating behavior [17]. In turn, disordered eating behaviors likely disrupt sleep-wake rhythms by delaying or reducing time in bed to accommodate disordered eating behavior (e.g., binge eating, driven exercise) or increasing physiological or psychological arousal resulting from emotional distress or physical discomfort [17]. Given the significance of disordered eating behaviors to health outcomes, the commonality of different disordered eating behaviors across diagnostic categories, and the potential public health relevance of intervening on individual disordered eating behaviors, further investigation of the relationships between individual transdiagnostic disordered eating behaviors and sleep is warranted. The purpose of the present study was to characterize the relationships between fasting, binge eating, self-induced vomiting, laxative or diuretic misuse, driven exercise, and sleep quality among a large online sample of adults. We predicted that engagement in disordered eating behaviors would be associated with poorer sleep quality, and sought to quantify the unique associations between individual behaviors and sleep quality.

## Method

### Participants

Individuals were eligible for the present study if they were 18 years or older and spoke English. There were no other exclusion criteria. Participants (*N* = 648) represented adults across the lifespan (from 18 to 80 years old)and primarily identified as female, non-Hispanic white, and heterosexual. Approximately half of the sample was classified as having overweight or obesity based on BMI which was calculated using self-reported height and weight. See Table 1 for participant characteristics.

### Procedures

Participants utilized Amazon’s Mechanical Turk (MTurk) online platform to complete a survey on eating and health attitudes between October 2017 and March 2018. The measures included in the present analyses were selected from the parent study because they were the only measures of sleep quality and disordered eating behavior frequency. Additional measures from the parent study not included in the present analyses are listed in Supplement 1. All surveys were presented in the same order for each participant: demographic information, eating/self-report height/weight, depression, and sleep. In general, MTurk produces reliable data, but there are potential threats to data quality [e.g., 19, 20]. A number of steps were taken to ensure data quality. First, to be included, participants needed to respond correctly to all five different types of validity questions (e.g., fill-in-the-blank, multiple choice). Of the participants who completed all the questionnaires, *n* = 157 were excluded for answering one or more questions incorrectly. We also inspected data for illogical or impossible response patterns. Participants were paid 0.50 cents. This study received approval from the Human Investigation Committee (i.e., IRB), and all participants provided active electronic informed consent by clicking one checkbox confirming they were at least 18 years of age and a second checkbox affirming “Yes, I provide consent to participate.”

### Measures

The Pittsburgh Sleep Quality Index (PSQI) [19] is a widely used 19-item self-report measure of sleep quality during the past month. The measure is comprised of seven subscales, including subjective sleep quality, sleep latency, sleep duration, sleep disturbance, use of sleep medication, sleep efficiency, and daytime dysfunction. Each subscale score ranges from 0 to 3, and subscales are summed to produce a global sleep quality score ranging from 0 to 21, with higher scores indicating worse sleep quality. Cronbach’s alpha for the current sample was 0.78.

The Eating Disorder Examination Questionnaire - Version 17 [20] is a self-report measure of eating disorder psychopathology during the past 28 days. Behaviors measured by this scale include objective binge eating episodes (“How many times have you eaten what other people would regard as an unusually large amount of food (given the circumstances)?” and “On how many of these times did you have a sense of having lost control over your eating (at the time you were eating)?”) and compensatory behaviors utilized as a means of weight control, including fasting (“Have you gone for long periods of time (8 waking hours or more) without eating anything at all in order to influence your shape or weight?”), self-induced vomiting, driven exercise (“Over the past 28 days, how many times have you exercised in a “driven” or “compulsive” way as a means of controlling your weight, shape or amount of fat, or to burn off calories?”), and laxative/diuretic misuse. These behaviors tend to have skewed distributions and a high zero-response so were examined dichotomously (i.e., either above *(1)* or below *(0)* subthreshold clinical cut-offs). Frequencies of objective binge eating, self-induced vomiting, driven exercise, and laxative/diuretic misuse were separately categorized as subthreshold if the frequency was two or more times in the past 28 days [18, 21]. For fasting, participants responded on a 0 (no days) to 6 (everyday) scale and scores of 2 or more (i.e., 6 or more days) were categorized as subthreshold [18, 21]. Self-report height and weight was collected as part of the EDE-Q.

The Patient Health Questionnaire − 2 (PHQ-2) [22] assesses the core symptoms of a major depressive episode in two self-report questions. Responses are scored on a 0 (“not at all”) to 3 (“nearly every day”) scale. The PHQ-2 has strong construct and criterion validity with independent structured interviews completed by trained professionals [22]. Higher scores suggest greater depression severity. Cronbach’s alpha for the current sample was 0.87.

### Statistical analysis

Pearson product-moment correlations were calculated between sleep and continuous variables (i.e., BMI, depression), Point-biserial correlations were calculated between sleep and dichotomous variables (i.e., disordered eating), and a Benjamini-Hochberg false discovery rate procedure was utilized. We conducted a hierarchical linear regression entering BMI and depression (variables known to be associated with problematic sleep) [23, 24] in step one and adding the five dichotomous disordered eating behavior variables (objective binge eating, fasting, self-induced vomiting, driven exercise, and laxative/diuretic misuse) in step two. PSQI global score was the outcome variable. Multicollinearity was not problematic in the present regression analyses (Variance Inflation Factors ranged from 1.02 to 1.55).

## Results

Table 1 presents descriptive data. The PSQI global score was significantly correlated with BMI (*r(648)* = 0.195, *p* < .001), depression (*r*(648) = 0.495, *p* < .001), objective binge eating frequency (*rpb*(648) = 0.297, *p* < .001), self-induced vomiting (*rpb*(648) = 0.107, *p* = .007), laxative/diuretic misuse (*rpb* (648) = 0.127, *p* = .001), fasting (*rpb*(648) = 0.259, *p* < .001), and driven exercise (*rpb(648)* = 0.098, *p* = .013). All significant correlations remained significant following a Benjamini-Hochberg false discovery rate procedure. Although a priori hypotheses were not proposed for specific dimensions of sleep quality measured by the PSQI subscales (e.g., sleep duration, sleep efficiency), bivariate correlations were conducted post hoc to explore the associations between disordered eating behaviors and PSQI subscale scores. No notable trends were observed (see Supplement 2). Table 2 presents results of the regression analyses, examining the presence or absence of subthreshold levels of disordered eating behaviors as correlates of sleep quality, after accounting for BMI and depression. The overall model was significant [R^2^ = 0.30, *F*(7, 640) = 31.21, *p* < .001], with objective binge eating and fasting, but not self-induced vomiting, driven exercise, or laxative/diuretic misuse, accounting for significant unique variance.

**Table 1:** Characteristics of the sample (N = 648)

	M *(SD)*	Range
Age	37.55 *(12.25)*	18–80
BMI	27.24 *(6.89)*	15.96–61.17
Disordered eating behaviors (monthly)^±^		
* Driven exercise episodes*	2.13*(5.34)*	0–49
* Self-induced vomiting episodes*	0.63*(4.49)*	0-100
* Objective binge eating episodes*	1.87*(4.16)*	0–30
* Laxative or diuretic episodes*	1.08*(4.91)*	0–60
Patient Health Questionnaire- 2	1.38*(1.65)*	0–6
Pittsburgh Sleep Quality Index Global Score	6.54*(4.00)*	0–19
	%	*n*
Sex		
* Female*	65.4	424
* Male*	34.3	222
* Missing*	0.3	2
BMI Category		
* Underweight*	2.6	17
* Healthy Weight*	38.3	248
* Overweight*	30.6	198
* Obese*	28.5	185
Sexual Orientation		
* Bisexual*	6.3	41
* Gay or Lesbian*	3.1	20
* Heterosexual*	90.1	584
* Other*	0.5	3
Education (highest degree)		
* Some middle school*	0.2	1
* Some high school*	0.5	3
* High school diploma*	6.9	45
* GED*	0.6	4
* Some college*	18.4	119
* Associate’s degree*	13.6	88
* College degree*	39.7	257
* Some graduate/professional*	4.2	27
* Graduate/professional degree*	16.0	104
Race and Ethnicity		
* American Indian or Alaska Native*	1.7	11
* Asian*	9.0	58
* Bi / Multiracial*	1.1	7
* Black*	7.1	46
* Native Hawaiian or Pacific Islander*	0.3	2
* White Hispanic*	7.4	48
* White, not Hispanic*	72.7	471
* Other racial/ethnic identity*	0.8	5
EDE-Q Fasting Threshold (2+)		
* Below 2 (fewer than 6 days per month)*	76.5	496
* At or above 2 (6 days or more per* *month)*	23.5	152
Objective binge episodes (monthly)		
* Less than 2*	70.2	455
* 2 or more*	29.8	193
Laxative or diuretic episodes (monthly)		
* Less than 2*	89.8	582
* 2 or more*	10.2	66
Driven exercise (monthly)		
* Less than 2*	77.5	502
* 2 or more*	22.5	146
Self-induced vomiting (monthly)		
* Less than 2*	92.7	601
* 2 or more*	7.3	47

± Fasting episodes were measured using acategorical scale, rather than as a count variable

**Table 2:** Hierarchical linear regression predicting sleep quality complaints measured by PSQI global score

	*R* ^2^	*R*^2^ Change	F (df)	Sig. F Change	B	SE	*p*	95% CI	VIF	
*Step 1*	0.263		114.82 *(2)*				**< 0.001**			
Constant					2.86	0.55	**< 0.001**	1.78, 3.94		
BMI					0.08	0.02	**< 0.001**	0.04, 0.12	1.02	
Depressive symptoms					1.16	0.83	**<0.001**	1.00, 1.32	1.02	
*Step 2*	0.300	0.038	39.21 *(7)*	**< 0.001**			**< 0.001**			
Constant					2.94	0.55	**< 0.001**	1.86, 4.01		
BMI					0.06	0.02	**0.004**	0.02, 0.10	1.06	
Depressive symptoms					1.04	0.09	**< 0.001**	0.87, 1.20	1.12	
Laxative or diuretic misuse					-0.29	0.54	0.594	-1.36, 0.78	1.55	
Driven exercise					0.15	0.35	0.66	-0.54, 0.84	1.22	
Self-induced vomiting					-0.60	0.62	0.330	-1.81, 0.61	1.46	
Objective binge eating					1.38	0.34	**< 0.001**	0.72, 2.03	1.34	
Fasting					1.04	0.35	**0.003**	0.35, 1.72	1.26	

Disordered eating behaviors were dummy-coded with 0 = no clinically significant behaviors or 1 = at or above minimum subthreshold behaviors. BMI = body mass index; PSQI = Pittsburgh Sleep Quality Index. B = unstandardized coefficient, VIF = Variance Inflation Factor

## Discussion

The purpose of the present study was to build upon the current literature examining the association between sleep and disordered eating. Our findings extend existing knowledge by investigating the extent to which individual transdiagnostic disordered eating behaviors were associated with sleep quality. Overall, results indicate that poor sleep quality was associated with all transdiagnostic disordered eating behaviors.

When included in multivariate models, objective binge eating and fasting were associated with more sleep quality complaints, whereas other compensatory behaviors were not. These findings are partially discrepant with previous work. For example, Nagata and colleagues [25] analyzed data from a cohort study of young adults, and found that fasting, other compensatory behaviors, and binge eating all predicted greater sleep disturbance seven years later. Some differences between these two studies, such as the older sample, cross-sectional design, and more comprehensive assessment of the present study, may account for this discrepancy.

The finding that objective binge eating is significantly related to sleep quality aligns with the conclusions of a recent meta-analysis which found that people who engage in binge eating reported substantially worse sleep quality compared to those who do not engage in binge eating [16]. It is plausible that certain behavioral features of binge eating contribute to worse sleep quality. For example, binge eating episodes are more likely to occur in the evenings [26, 27], which could impair ability to fall asleep. Binge eating behavior also often is associated with feelings of disgust, depression, and guilt [28], and such negative affective states may disrupt sleep quality [29]. In turn, poor sleep quality may increase the likelihood of engagement in binge eating through multiple pathways. For example, insufficient sleep is known to influence risk factors for binge eating behavior such as diminished executive function or heightened negative affect [29, 30]. Thus, the present findings align with existing literature on binge eating, though temporal relationships between sleep and binge eating behavior are not yet clearly understood.

The extant literature is less consistent with regard to the relationships between compensatory behaviors and sleep, but it may be that the specific associations vary across clinical contexts. For example, studies comparing subtypes of anorexia nervosa (i.e., restrictive vs. binge/purge subtypes) found that the binge/purge subtype (i.e., binge eating and compensatory behaviors) was associated with greater sleep disturbance, whereas the restrictive subtype (i.e., fasting) was not [31, 32]. Patients with bulimia nervosa, which also is characterized by compensatory behaviors, report elevated levels of subjective sleep disturbance, but show minimal sleep impairments when measured by objective sleep assessment [33, 34]. It may be that the relationship between sleep and compensatory behaviors is dependent upon the presence of other disordered eating behaviors (i.e., binge eating, fasting), resulting in a lack of clarity in the extant literature. Our data suggest that compensatory behaviors are not significantly associated with sleep quality after accounting for BMI, depression, and other disordered eating behaviors. Alternatively, in our non-clinical sample, it may be that there was not sufficient variance in self-induced vomiting or laxative/diuretic use to capture substantial sleep impairment related to these less common behaviors.

While the current paper contributes to the literature, it also is important to consider the limitations. First, the sample was an online convenience sample. Despite the use of best practices for online data, it is possible that some respondents may have provided poor quality data [35, 36]. The non-clinical nature of the sample may limit our ability to detect effects. Second, the sample was predominantly white, female, and college educated, which limits generalizability of findings. Third, these data were collected prior to COVID, and the results may underestimate current community rates of problematic eating and sleep behaviors [37]. Fourth, both sleep and disordered eating behaviors were assessed using self-report measures. Although the measures selected for this study are valid and widely-used, they are subject to more potential bias than clinical or objective measures. Fifth, due to somewhat low frequencies, use of diuretics and laxatives was combined; therefore, differences in relationships with these variables may have been overlooked. Sixth, participants completed the measures as part of a larger survey study, it is unknown if completing these other measures impacted their responses to the current measures in any way or similarly, if completing questionnaires about eating and weight prior to a depression assessment impacted responses. Finally, as this study was cross-sectional, it is not possible to establish the causality of these associations and investigate temporal bidirectional effects. In future studies, designs that use longitudinal measurement may be better suited to capture these dynamic relationships.

## Conclusions

Altogether, this study contributes novel findings regarding the independent associations between different disordered eating behaviors and sleep quality complaints in a large, online sample of adults. Improved understanding of these specific relationships is important, particularly for non-clinical samples, as most disordered eating behaviors occur across diagnostic categories and are common among individuals with subclinical symptoms. Our findings suggest that individuals who report, at minimum, subclinical binge eating and fasting behaviors may be particularly vulnerable to poor sleep quality, which is problematic because in addition to the negative physical and psychological effects of poor sleep, sleep disturbance has the potential to further exacerbate disordered eating behavior. Future research could clarify these effects with the use of prospective or intensive longitudinal designs to establish temporal precedence between disordered eating behaviors and sleep and a more rigorous assessment of both disordered eating behaviors and sleep disturbance. In addition, evaluation of disordered eating behaviors in cycles (e.g., binge/fast cycles) rather than in isolation could identify more distinct sleep profiles across clinical presentations.

### Electronic supplementary material

Below is the link to the electronic supplementary material.

Supplementary Material 1

Supplementary Material 2

## Data Availability

No datasets were generated or analysed during the current study.

## Abbreviations

BMI: Body Mass Index
MTurk: Mechanical Turk
PSQI: Pittsburgh Sleep Quality Index
PHQ-2: Patient Health Questionnaire-2
