# Supplementary material for: Engagement in binge eating and fasting associated with poorer sleep quality in an online sample of adults
Source: J Eat Disord. 2024 Jun 10;12:76. doi: 10.1186/s40337-024-01028-6 (PMC11165844; doi:10.1186/s40337-024-01028-6)
Supplement: Supplementary file 1 — Supplementary Material 1 [file 40337_2024_1028_MOESM1_ESM.docx]

**Supplement 1**

The analyses presented in this manuscript were conducted using a subset of measures from a larger cross-sectional online parent study. Please see the following references for the additional questionnaires included in the parent study, but not used for the present manuscript:

1. Barnes, R. D., Ivezaj, V., & Grilo, C. M. (2016). Food Thought Suppression Inventory: Test–retest reliability and relationship to weight loss treatment outcomes. *Eating behaviors*, *22*, 93-95.
2. Gearhardt, A. N., Corbin, W. R., & Brownell, K. D. (2009). Preliminary validation of the Yale food addiction scale. *Appetite*, *52*(2), 430-436.
3. Durso, L. E., & Latner, J. D. (2008). Understanding self‐directed stigma: development of the weight bias internalization scale. *Obesity*, *16*(S2), S80-S86.
4. Victor, S. E., & Klonsky, E. D. (2016). Validation of a brief version of the difficulties in emotion regulation scale (DERS-18) in five samples. *Journal of psychopathology and Behavioral Assessment*, *38*, 582-589.
5. Sheehan DV. The Anxiety Disease. New York, NY, USA: Charles Scribners Sons; 1983.
6. Palmeira, L., Cunha, M., Pinto-Gouveia, J., Carvalho, S., & Lillis, J. (2016). New developments in the assessment of weight-related experiential avoidance (AAQW-Revised). *Journal of Contextual Behavioral Science*, *5*(3), 193-200.
7. Blomquist, K. K., Roberto, C. A., Barnes, R. D., White, M. A., Masheb, R. M., & Grilo, C. M. (2014). Development and validation of the eating loss of control scale. *Psychological assessment*, *26*(1), 77.
8. White, M. A., Whisenhunt, B. L., Williamson, D. A., Greenway, F. L., & Netemeyer, R. G. (2002). Development and validation of the food‐craving inventory. *Obesity research*, *10*(2), 107-114.
9. Vaz, F. J., Alcaina, T., & Guisado, J. A. (1998). Food aversions in eating disorders. *International journal of food sciences and nutrition*, *49*(3), 181-186.
10. Allison, D. B., Basile, V. C., & Yuker, H. E. (1991). The measurement of attitudes toward and beliefs about obese persons. *International Journal of Eating Disorders*, *10*(5), 599-607.
11. Dickson-Spillmann, M., Siegrist, M., & Keller, C. (2011). Development and validation of a short, consumer-oriented nutrition knowledge questionnaire. *Appetite*, *56*(3), 617-620.
12. Watkins, C. M., Lartey, G. K., Golla, V., & Khubchandani, J. (2008). Workers perception: Environmental factors influencing obesity at the workplace. *American Journal of Health Studies*, *23*(2).
