# Supplementary material for: Engagement in binge eating and fasting associated with poorer sleep quality in an online sample of adults
Source: J Eat Disord. 2024 Jun 10;12:76. doi: 10.1186/s40337-024-01028-6 (PMC11165844; doi:10.1186/s40337-024-01028-6)
Supplement: Supplementary file 2 — Supplementary Material 2 [file 40337_2024_1028_MOESM2_ESM.docx]

**Supplement 2**

Point-biserial correlations were conducted as an exploratory, post hoc analysis to estimate the associations between individual subscales of the Pittsburgh Sleep Quality Index (PSQI) and the presence or specific disordered eating behaviors. Results are presented in the table below.

| **Point-biserial Correlations between Sleep Quality Subscales and Disordered Eating Behaviors** | | | | | |
| --- | --- | --- | --- | --- | --- |
| **Variable** | Restriction | Vomiting | Laxative and Diuretic Use | Objective Binge Episodes | Compensatory Exercise |
| Subjective Sleep Quality | .16 ** | .02 | .04 | .12** | .04 |
| Sleep Latency | .14** | .11** | .05 | .21** | .10* |
| Sleep Duration | .13** | -.02 | .02 | .10* | .04 |
| Sleep Efficiency | .12** | .00 | .00 | .17* | .05 |
| Sleep Disturbance | .22** | .16** | .18** | .31** | .16** |
| Use of Sleep Medication | .19** | .08* | .14** | .20* | .02 |
| Daytime Dysfunction | .25** | .15** | .15** | .27** | .06 |

Sleep quality subscales represent sleep quality complaints, with higher scores indicating worse sleep quality, Disordered eating behaviors are count variables indicating total number over the past 28 days, except for fasting, which is a categorical variable with categories indicating ranges of days in which fasting occurred; **Correlation is significant at the 0.01 level (2-tailed); *Correlation is significant at the 0.05 level (2-tailed).
